# Lassa fever and Argentine hemorrhagic fever treatment in guinea pigs using broad-spectrum cap-dependent endonuclease inhibitors

**DOI:** 10.1128/mbio.00880-26

**Published:** 2026-08-17

**Authors:** Junki Maruyama, Satoshi Taniguchi, Takeshi Saito, Shintaro Yamada, Rachel A. Reyna, Ruchi Paroha, Daiki Kobayashi, Gavriella Siman-Tov, Christine Click, Kirsten Littlefield, Shinsuke Toba, Yoshiyuki Taoda, Misato Shibazaki, Cheng Huang, Michihito Sasaki, Yasuko Orba, Hirofumi Sawa, Akihiko Sato, Slobodan Paessler

**Affiliations:** 1 Department of Pathology, The University of Texas Medical Branch 12338, Galveston, Texas, USA; 2 Institute for Human Infections and Immunity 551582, Galveston, Texas, USA; 3 Department of Molecular Biology and Biochemistry, The University of Texas Medical Branch 198643, Galveston, Texas, USA; 4 Department of Microbiology and Immunology, The University of Texas Medical Branch 547647, Galveston, Texas, USA; 5 Shionogi and Co Ltd 13350 , Osaka, Japan; 6 Division of Molecular Pathobiology, International Institute for Zoonosis Control, Hokkaido University 12810, Sapporo, Japan; 7 Institute for Vaccine Research and Development, Hokkaido University 12810, Sapporo, Japan; 8 International Collaboration Unit, International Institute for Zoonosis Control, Hokkaido University 12810, Sapporo, Japan; University of Pennsylvania, Philadelphia, Pennsylvania, USA

**Keywords:** arenavirus, Lassa virus, Junin virus, antiviral, hemorrhagic fever, cap-dependent endonuclease

## Abstract

The class *Bunyaviricetes* encompasses several highly pathogenic viruses that cause lethal hemorrhagic fevers. Due to their limited prevention and treatment options and high pathogenicity, these viruses require handling in biosafety level-4 facilities. Within the bunyaviruses, arenaviruses are particularly notable for their pathogenicity and ability to cause severe hemorrhagic disease in humans. The cap-dependent endonuclease (CEN) is a unique and crucial enzyme involved in the replication cycle of these viruses. As humans do not possess a similar enzyme, CEN represents an ideal target for antiviral drug development with reduced risk of side effects. Recently, we identified a promising CEN inhibitor (CENi) demonstrating potent inhibition of virus replication. In this manuscript, we demonstrate the successful therapeutic efficacy of CENis against Lassa fever and Argentine hemorrhagic fever virus infections in guinea pig models of lethal hemorrhagic fever. In addition, we identified several CENis with antiviral activity against other highly pathogenic arenaviruses. These findings further support the potential of CENis as therapeutic agents for arenavirus infections that cause severe and often lethal hemorrhagic fever. Collectively, our results suggest that CENis are promising candidates for pan-arenavirus therapy and may also have broader utility against other CEN-containing viruses for which no approved antiviral treatments currently exist.

**IMPORTANCE:**

Arenaviruses, such as Lassa virus and Junin virus, cause severe hemorrhagic fevers in humans and are associated with high mortality rates and limited treatment options. Because of their pathogenicity and potential for outbreaks, these viruses represent an important global health threat. The viral cap-dependent endonuclease (CEN) plays a critical role in arenavirus replication by enabling the cap-snatching process required for viral mRNA synthesis. Notably, mammalian cells lack a comparable enzyme, making CEN an attractive target for antiviral drug development. In this study, we demonstrate that inhibitors targeting the arenavirus CEN effectively suppress viral replication and provide therapeutic protection in animal models of lethal Lassa virus and Junin virus infections. We also identify compounds with antiviral activity against multiple highly pathogenic arenaviruses. These findings establish CEN inhibition as a promising strategy for the development of broad-spectrum antivirals against arenaviruses that cause life-threatening hemorrhagic fevers.

## INTRODUCTION

The family *Arenaviridae* includes several viruses that cause lethal hemorrhagic fevers in humans, such as Lassa virus (LASV), Lujo virus (LUJV), Junin virus (JUNV), Machupo virus (MACV), Guanarito virus (GTOV), Sabia virus (SABV), and Chapare virus (CHAPV). These pathogens represent a significant public health threat in endemic regions and potentially worldwide via imported cases (1, 2). Moreover, the highly pathogenic arenaviruses are listed as Risk Group-4 pathogens and classified as Category A Priority Pathogens, due to their high risk to public health and national security. Lassa fever (LF), caused by LASV, is one of the most serious public health threats in endemic regions due to its high morbidity and mortality rates (3, 4). Although the real numbers of LASV infections are not known, LF is a significant threat to public health in the endemic regions of West Africa, such as Nigeria, Guinea, Liberia, and Sierra Leone, and up to 500,000 potential cases of LASV infection are annually proposed (5). For example, Nigeria is a hotspot for LASV infection, with the largest number of annual confirmed cases being 1,189 in 2020 (6). Furthermore, the recent 2018 outbreak in Nigeria is considered unprecedented, with 633 confirmed cases and a case fatality rate of 27% (7). The general case fatality rate for hospitalized cases ranges from 15% to 70% depending on the outbreak, and an estimated 37.7 million people might be at risk of infection with LASV annually (8–10). Likewise, the New World arenaviruses (NWAs), JUNV, MACV, GTOV, SABV, and CHAPV cause South American hemorrhagic fevers characterized by hemorrhagic and neurologic manifestations with high mortality (11). Although the majority of hemorrhagic fevers in South America are caused by infections with JUNV or MACV, human cases infected with GTOV, SABV, and CHAPV have also been reported in recent years (12–14). Although the endemic distribution of arenavirus infections is closely linked to the geographic range of their natural rodent reservoirs, there is growing concern that human economic activities may be expanding the range of these reservoirs and increasing human exposure. As a result, arenavirus infections may continue to emerge and re-emerge in new geographic areas (15). However, effective therapeutic drugs against these pathogenic arenaviruses are very limited, and thus, the development of potent drugs against these viral diseases is a priority for global public health.

The viruses belonging to the class *Bunyaviricetes* have a cap-dependent endonuclease (CEN) as an essential viral enzyme that initiates viral transcription by RNA-dependent RNA polymerase (RdRp), as reported for orthomyxoviruses (16). CEN is an ideal target for antiviral drugs since it is a virus-specific enzyme that human cells do not possess, thereby reducing potential side effects. In fact, a CEN inhibitor (CENi), baloxavir marboxil, has been approved as an antiviral drug against influenza in Japan and the United States (17). Recently, we reported a CENi with broad-spectrum antiviral inhibitory effects *in vitro* and *in vivo* against numerous members of the family *Arenaviridae* (18). In this paper, we evaluated the antiviral activity of CENi compounds against highly pathogenic arenaviruses *in vitro* and assessed its *in vivo* efficacy in guinea pig models challenged with LASV or JUNV, the principal arenaviruses of concern in the Old World and New World, respectively. Our findings demonstrate that CENi compounds are promising candidates for the development of pan-arenavirus therapies.

## RESULTS

### *In vitro* antiviral efficacy of CENi compounds against arenavirus infections

**Fig 1:**
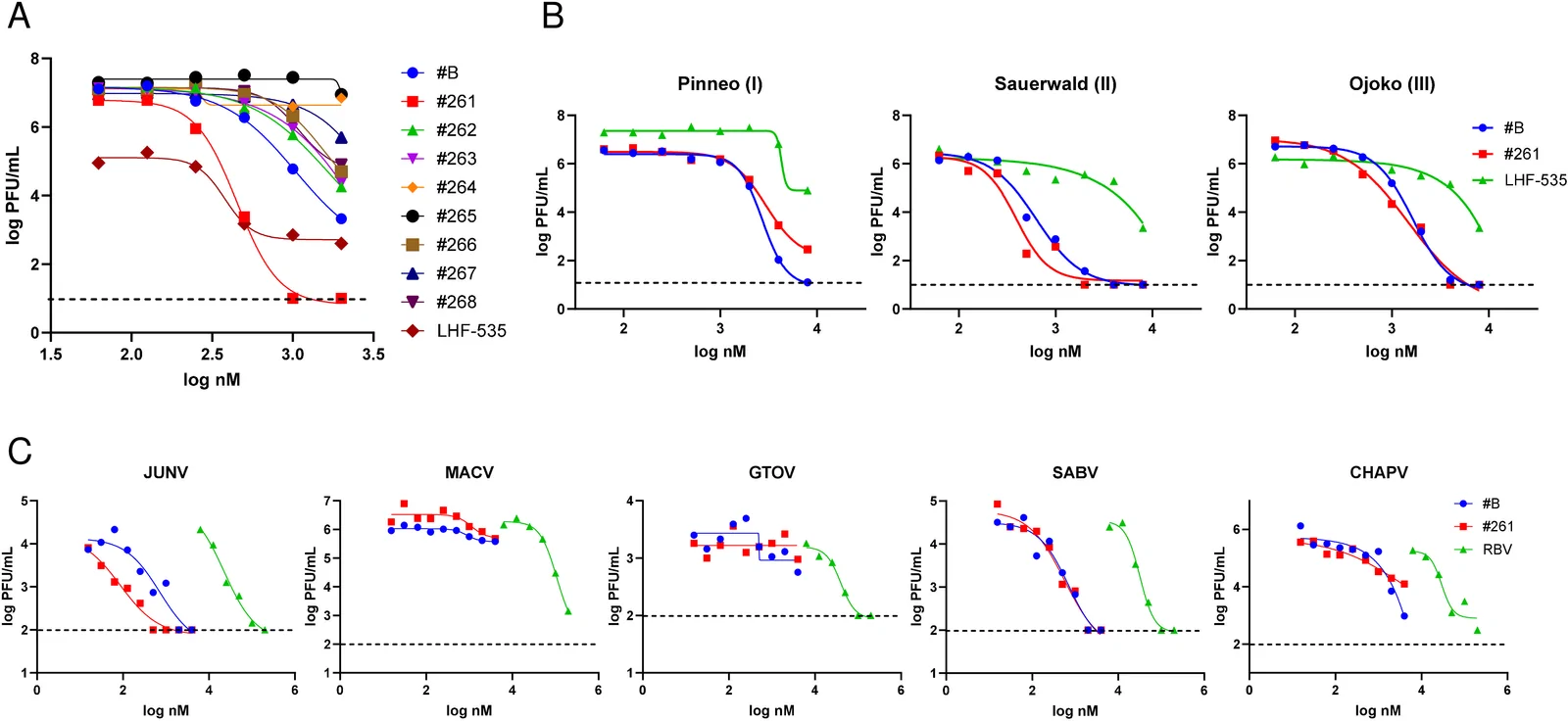
Virus replication inhibition activity of CENi compounds against arenaviruses. (**A**) Screening of CENi compounds structurally related to compound #B (18) was performed by virus replication inhibition assay with LASV LF2384 (lineage IV). LHF-535 (19) was used as a positive control. (**B**) Antiviral activities of compound #B, #261, and LHF-535 against LASV Pinneo, Sauerwald, and Ojoko (lineages I, II, and III, respectively) were measured by virus replication inhibition assay. (**C**) Antiviral activities of compounds #B and #261 against JUNV (Romero), MACV (Chicava), GTOV (S-26764), SABV (SPH114202), and CHAPV (200401071) were measured by virus replication inhibition assay. Ribavirin (RBV) was used as a positive control of treatment. Each plot shows the mean of triplicate. The dose-response sigmoidal curve was determined by four-parameter logistic curve fitting. The broken lines indicate the limit of detection (<10 PFU/mL for panels **A and B**; <100 PFU/mL for panel **C**).

Recently, we identified a CENi, compound #B, that has potent antiviral activity against multiple bunyaviruses (18). We evaluated the virus replication inhibition activity of eight additional compounds with structural similarity to compound #B against LASV (LF2384; lineage IV) (Fig. 1A). As a result, we found that compound #261 had potent antiviral activity (IC_50_ = 451.4 [95% CI: 354.1 to 566.8] nM). Notably, we did not detect any infectious virus from the samples treated with more than 1 µM of compound #261. We further investigated the antiviral activity against LASVs belonging to lineages I–III (Fig. 1B). Compounds #B and #261 showed potent virus replication inhibition against LASVs (lineages I–III), whereas LHF-535 was less effective against LASVs except lineage IV (Table S1). In addition, we evaluated the antiviral activity of CENi compounds against JUNV, MACV, GTOV, SABV, and CHAPV, all of which are causative agents of South American hemorrhagic fevers (Fig. 1C). Compound #B or #261 showed virus replication inhibition against JUNV, SABV, and CHAPV with 10–100 times lower IC_50_ values compared with ribavirin (RBV) (Table S1). Unfortunately, neither compound #B nor #261 was effective against MACV or GTOV.

### *In vivo* efficacy of #261 treatment in guinea pigs infected with LASV

**Fig 2:**
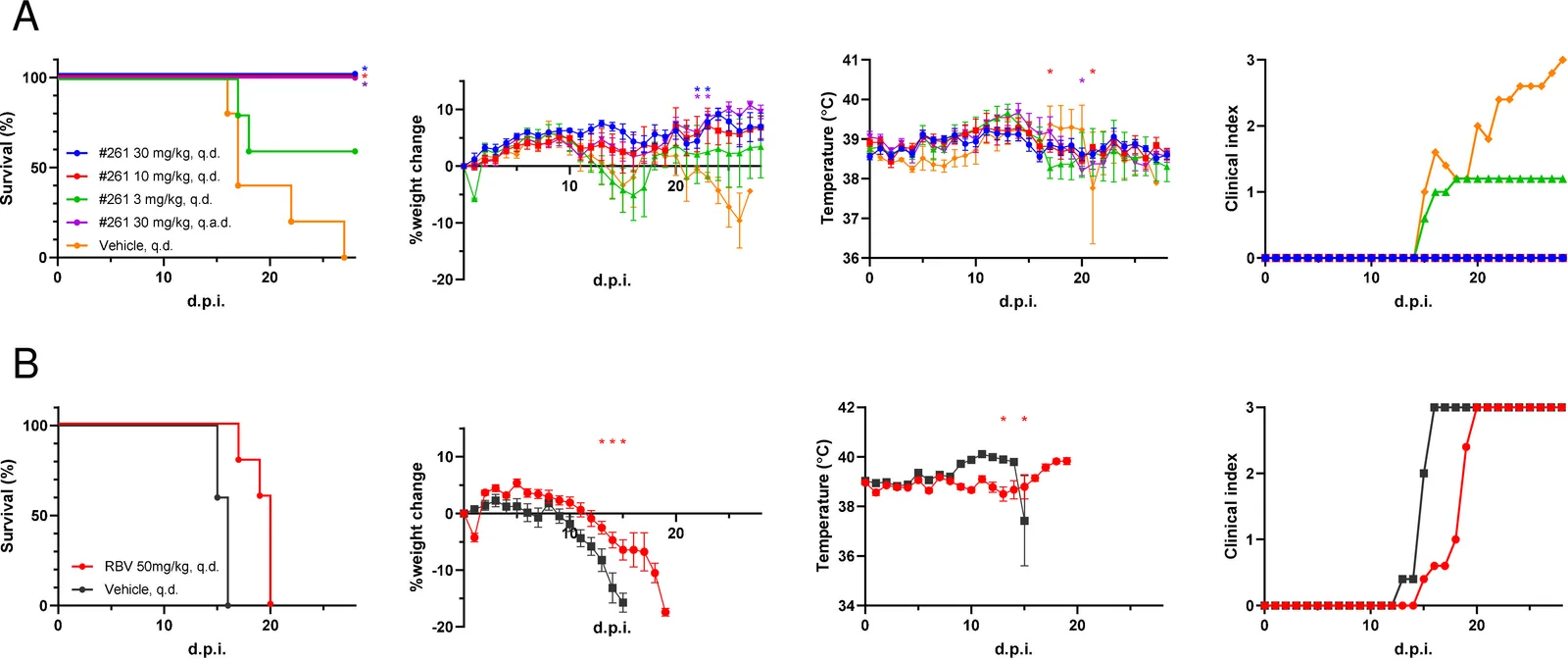
*In vivo* efficacy of CENi treatment in guinea pigs infected with LASV. Guinea pigs (*N* = 5) were treated with compound #261, ribavirin (RBV), or each vehicle starting at 2 d.p.i. Survival rate, body weight change, body temperature, and clinical index of guinea pigs treated with compound #261 (**A**) or RBV (**B**) were shown. Each vehicle was used as a negative control of treatment. Means and standard errors were shown for body weight change and body temperature. Statistical differences for survival compared with the vehicle control group were detected by the log-rank test followed by Bonferroni correction (*P < 0.01). Statistical differences for body weight/temperature change compared wirh the vehicle control group were detected by two-way ANOVA followed by Dunnett’s multiple comparison test (*P < 0.05).

**Fig 3:**
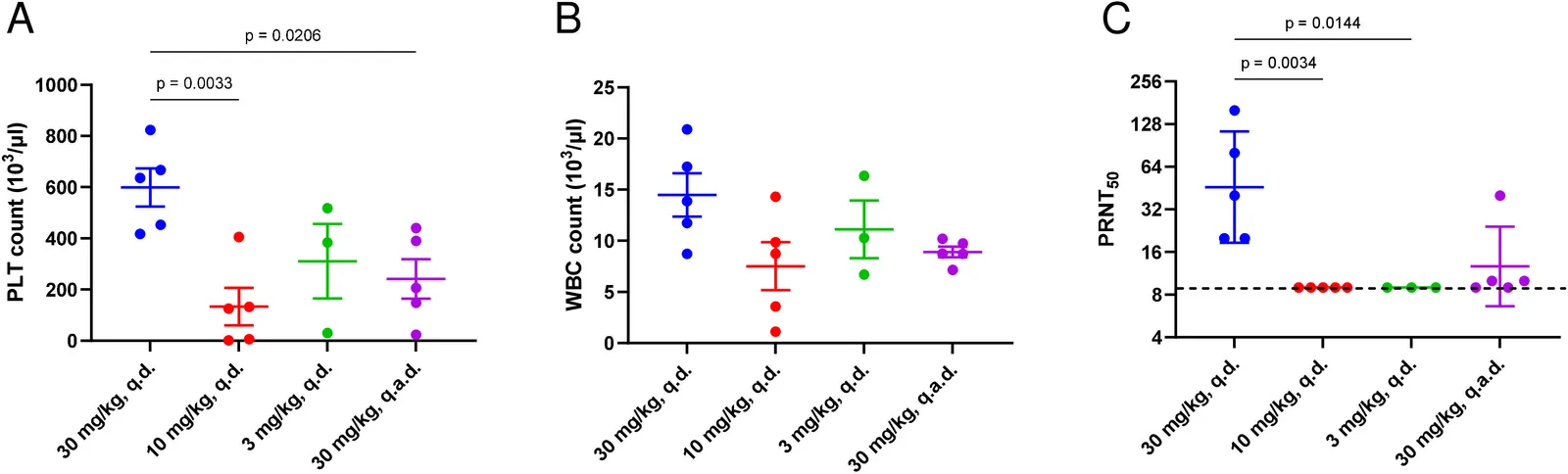
CBC and antibody response in guinea pigs infected with LASV. (**A**) Platelets and (**B**) white blood cells were counted with blood samples from survivors from LASV challenge. Means and standard errors were presented. (**C**) Neutralizing antibody titers in serum samples were measured by plaque reduction neutralizing test. Geometric means and geometric standard deviations were presented. The broken line indicates the limit of detection (<10). Statistical differences were detected by one-way ANOVA following Dunnett’s multiple comparison test.

**Fig 4:**
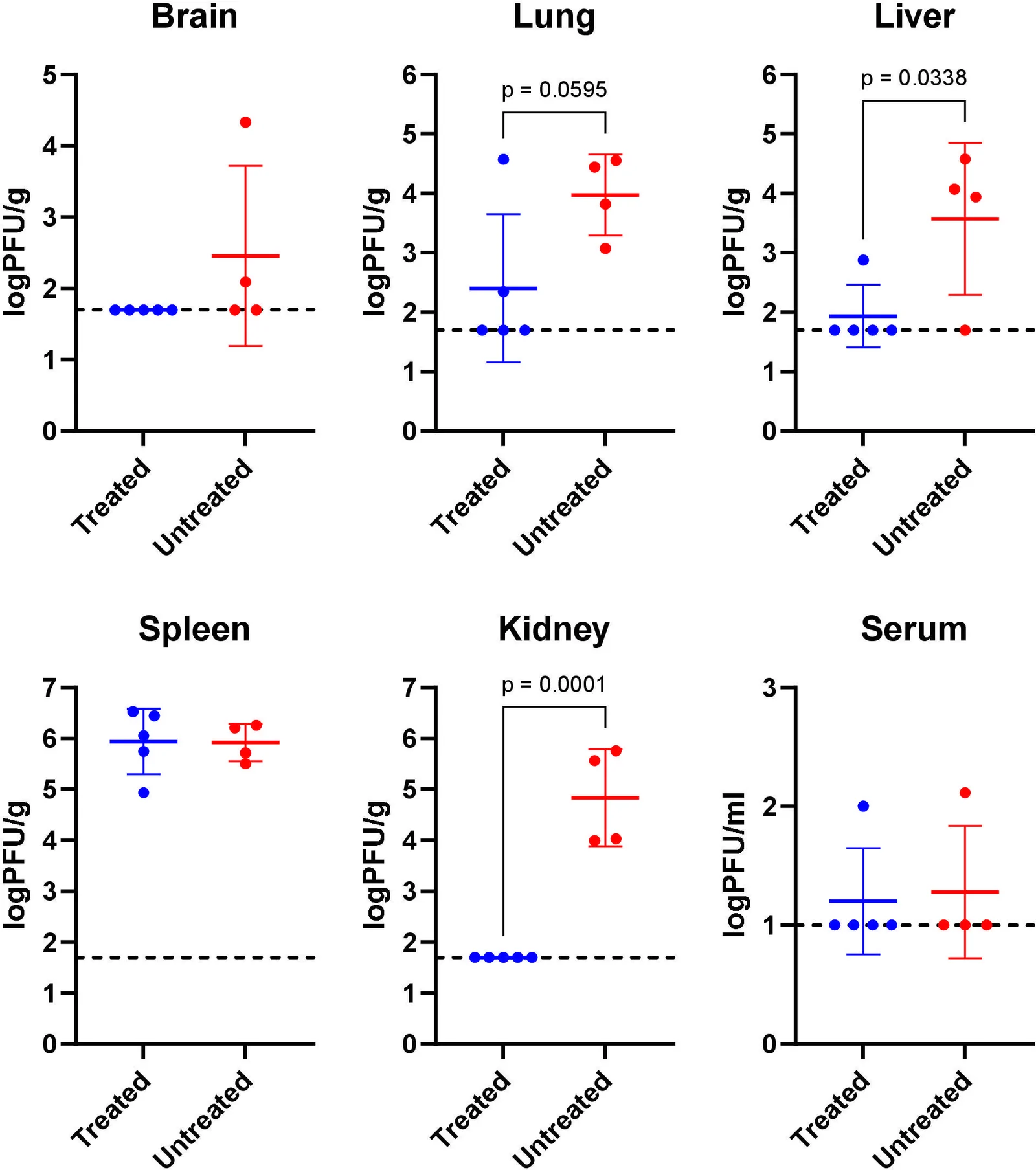
LASV dissemination in guinea pigs treated with #261 or untreated. Organ or blood samples were collected from guinea pigs infected with LASV in Treated (compound #261 30 mg/kg, q.d) or Untreated (vehicle control, PEG-400, q.d.) at 11 d.p.i. Means and standard errors were presented. The broken line indicates the limit of detection (organs: <1.70 PFU/g, serum: <1.00 PFU/mL). Statistical differences were detected by Welch’s *t*-test.

We evaluated the treatment efficacy of compound #261 in the guinea pig model of Lassa fever. Hartley guinea pigs were challenged with 10^3^ plaque forming units (PFU) of LASV (LF2384), approximately 100 median lethal doses (LD_50_), and then treated with compound #261, RBV, or each vehicle control daily (q.d.) or once every two days (q.a.d.) for 14 days, starting at 2 d.p.i. All guinea pigs in 30 and 10 mg/kg, q.d. or 30 mg/kg, q.a.d. groups survived lethal LASV challenge (*P* = 0.0018, significant level α = 0.01 compared with the vehicle control group) (Fig. 2A). No visible disease signs were observed in these animals throughout the study. The guinea pigs in 3 mg/kg, q.d. group showed 60% survival (*P* = 0.0703 compared with the vehicle control group), whereas all animals in the vehicle control group succumbed to the disease by 27 d.p.i. (Fig. 2A). Visible disease signs, including unkempt fur, hunched posture, and lethargy, were observed in animals that succumbed to the disease. Of note, although ribavirin (RBV) treatment delayed the onset of LASV infection, none of the animals treated with RBV (50 mg/kg, q.d.) survived lethal LASV infection (Fig. 2B). At the time of necropsy, no specific pathologic changes were observed except for small spleens and pale livers on gross pathology in animals that succumbed to the disease, consistent with the previous study (20). Blood samples were collected from survivors at the end of the study (28 d.p.i.) for the complete blood cell count (CBC) (Table S2). Platelet counts in animals of 30 mg/kg, q.d. group were in normal range, whereas animals in the other groups showed thrombocytopenia (Fig. 3A). White blood cell (WBC) counts showed the same trend (Fig. 3B). Interestingly, survivors in 30 mg/kg, q.d. group showed higher neutralizing antibody titers (Fig. 3C). This result suggests that the treatment successfully eliminated LASV, resulting in the prevention of lymphopenia and allowing for a robust induction of antibody response. Virus disseminations were assessed in the samples from compound #261 30 mg/kg, q.d. group (Treated, *n* = 5) or vehicle control group (Untreated, *n* = 4) at 11 d.p.i. (Fig. 4). Virus titers in livers and kidneys from Treated group are significantly lower than those from Untreated group. In addition, virus titers in lungs had lower trends in Treated group compared with Untreated group. In contrast, virus titers in spleens and sera were comparable. Of note, any infectious viruses were not detected from brain or kidney samples from Treated group. CBC results at 11 d.p.i. revealed that the decrease in neutrophils was observed in Untreated group. However, thrombocytopenia was observed in Treated (4/5) and Untreated (3/4) groups (Table S3).

### *In vivo* efficacy of #261 treatment in guinea pigs infected with JUNV

**Fig 5:**
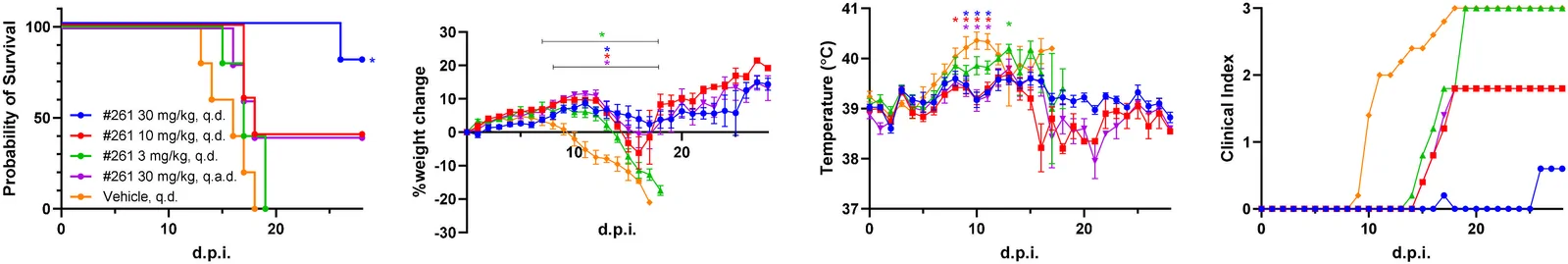
*In vivo* efficacy of CENi treatment in guinea pigs infected with JUNV. Guinea pigs (*N* = 5) were treated with compound #261 or vehicle starting at 2 d.p.i. Survival rate, body weight change, body temperature, and clinical index of guinea pigs treated with compound #261 were shown. Means and standard errors were shown for body weight change and body temperature. Statistical differences for survival compared with the vehicle control group were detected by log-rank test followed by Bonferroni correction (*P < 0.01). Statistical differences for body weight/temperature change compared with the vehicle control group were detected by two-way ANOVA followed by Dunnett’s multiple comparison test (*P < 0.05).

We also evaluated the treatment efficacy of compound #261 in the guinea pigs infected with JUNV. Hartley guinea pigs were challenged with JUNV (Romero) and then treated with compound #261 using the same protocol as in the LASV study. The guinea pigs in 30 mg/kg, q.d. group showed 80% survival from lethal JUNV challenge (*P* = 0.0039, significant level α = 0.01 compared with the vehicle control group) (Fig. 5). The guinea pigs in 10 mg/kg, q.d. or 30 mg/kg, q.a.d. groups showed 40% survival (*P* = 0.0487 or *P* = 0.0812, respectively, significant level α = 0.01 compared with the vehicle control group). All guinea pigs treated with 3 mg/kg, q.d., or vehicle succumbed to the disease by 18 or 17 d.p.i., respectively. Of note, 5 of the 12 animals that succumbed to the disease in the compound #261-treated groups exhibited neurological symptoms such as generalized or limb paralysis, whereas all animals in the vehicle-treated group succumbed to acute systemic disease. The CBC results showed lymphopenia and thrombocytopenia in all groups (Table S4). More hemorrhagic signs were observed in the vehicle control group based on gross pathology at necropsy (Table S5).

### Additional screening to identify CENis that inhibit MACV and GTOV replication

**Fig 6:**
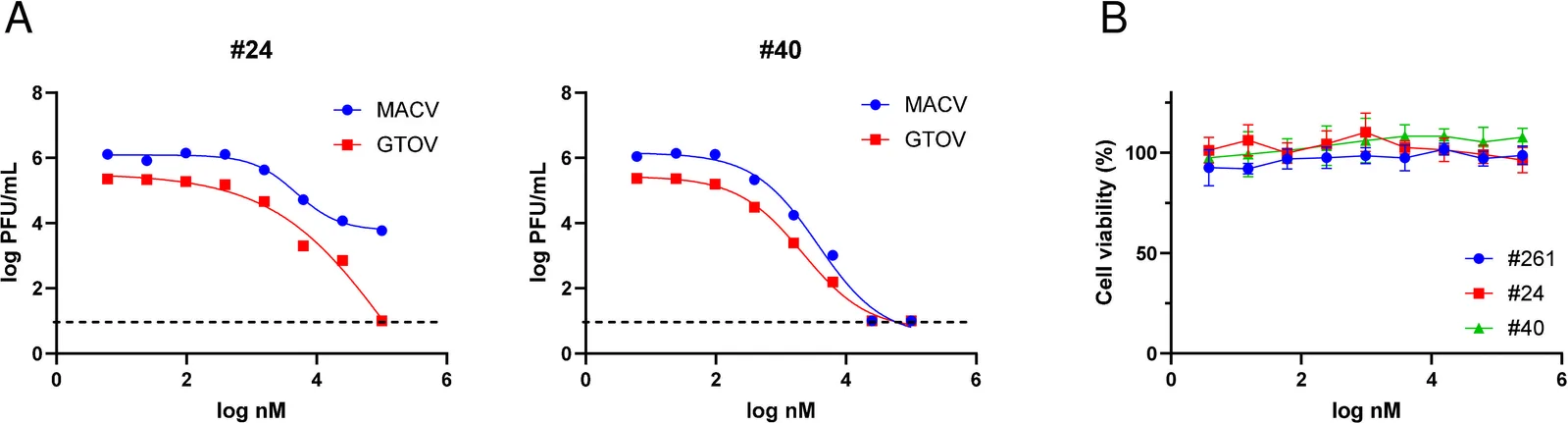
Virus replication inhibition activity of CENi compounds against MACV and GTOV. (**A**) Antiviral activities of compounds #24 and #40 against MACV (Chicava) and GTOV (VINH-95551) were measured by virus replication inhibition assay. Each plot shows the mean of triplicate. The dose-response sigmoidal curve was determined by four-parameter logistic curve fitting. The broken lines indicate the limit of detection (<10 PFU/mL). (**B**) Cell cytotoxisity of compounds #261, #24, and #40 were measured by cell viability assay. Each plot shows the mean of triplicate. Error bars show standard errors.

To identify CENis that have a potent inhibitory effect against MACV and GTOV replication, 52 small compounds structurally related to compound #261 were used for virus replication inhibition assay (Table S6). We identified that #24 and #40 inhibited MACV and GTOV well (Fig. 6A). It is worth noting that #40 exhibited potent virus replication inhibition against MACV (IC_50_ = 3,705 [95% CI: 2,775–5,115] nM) and GTOV (IC_50_ = 2,160 [95% CI: 1,798–2,616] nM) with complete inhibition at more than 25 µM. It is worth noting that these compounds did not exhibit cytotoxicity until 250 µM, as was the case for compound #261 (Fig. 6B). The 50% cytotoxic concentration (IC_50_) values are >250 µM.

## DISCUSSION

Arenaviruses are typical zoonotic pathogens, and their diversity is based on evolution in their specific natural rodent hosts (21). LASVs are divided into at least seven lineages (22, 23). The recent discovery of new LASV lineages, which is based on the improved surveillance activities in endemic areas, suggests that the diversity of LASVs is higher than previously thought. To date, five species of arenaviruses are known to cause hemorrhagic fever in the New World (NWAs). Although the majority of South American hemorrhagic fevers are thought to be caused by JUNV or MACV, cases of CHAPV or SABV-like novel virus infections have recently been reported (13, 24). The true number of NWA infections remains unknown due to poor surveillance in endemic areas. Therapeutic methods with broad-spectrum capabilities are essential for the development of a control strategy against future outbreaks caused by arenavirus infections.

LHF-535 is one of the most advanced antiviral candidates against Lassa fever to date (19, 25) as it has already completed phase I clinical trials (26). LHF-535 is as effective as compound #261 against LASV belonging to lineage IV. However, LHF-535 has less viral replication inhibitory activity against LASV lineage I-III compared with compound #261. Nevertheless, LHF-535 and compound #261 have different mechanisms of action, so combination treatment of these antiviral candidates may improve treatment efficacy against LF in the future.

Some antiviral candidates against arenavirus infections have already been reported, such as ribavirin and favipiravir (27, 28). These antiviral candidates exhibit broad-spectrum activity by inhibiting viral replication through their action as RdRp inhibitors similar to CENi. However, it is important to note that compounds such as ribavirin or favipiravir are nucleotide analogs that also impact the physiology of mammalian cells, raising concerns about potential side effects. For instance, favipiravir is not safe for treatment in infants or during pregnancy. In contrast, our focus on CENi targets a virus-specific enzyme, the cap-dependent endonuclease, which is not present in humans and should allow for a safer drug development strategy. An illustrative example is the FDA-approved CENi used against the influenza virus, baloxavir marboxil. This medication has been approved for all people aged 12 and older and for children aged 5–12 with no chronic conditions and is successfully employed in influenza patients without any significant reports of severe side effects, underscoring the high selectivity and safety profile of CENi as an ideal antiviral candidate. Additionally, our results showed that ribavirin treatment delayed the onset of LASV infection, but the body condition suddenly deteriorated once treatment was stopped, resulting in a lethal outcome of the disease. A similar phenomenon has been reported in JUNV-infected guinea pigs treated with ribavirin (29).

Compound #261 treatment successfully protected guinea pigs from lethal LASV challenge. Notably, guinea pigs treated with 30 mg/kg, q.d. of compound #261 did not show lymphopenia and thrombocytopenia, and a neutralizing antibody response was observed. LASV infection is known to suppress host immune response, resulting in a diminished T-cell and humoral immunity (30, 31). Our results indicate that treatment with compound #261 either eliminated LASV during the early stage of infection or prevented systemic virus dissemination. This likely reduced the immunosuppressive effects of LASV infection, allowing the host immune response to develop more effectively and contribute to viral clearance. At 11 d.p.i., virus titers in the spleen were similar between treated and untreated animals, indicating that treatment initiated at 2 d.p.i. did not completely prevent replication in the spleen, which is a primary target organ for LASV. However, compound #261 appeared to block dissemination to other organs, thereby limiting disease progression and improving survival (20, 32).

The *in vivo* efficacy of compound #261 against JUNV was lower than that observed against LASV. Animals that succumbed to JUNV infection frequently developed neurological signs. Because these animals were euthanized upon reaching humane endpoint criteria, it remains unclear whether the neurological manifestations would have been transient or permanent. These findings suggest that compound #261 reduced JUNV replication during the early phase of infection, thereby limiting systemic spread and preventing severe hemorrhagic disease. However, the compound appeared to be less effective at eliminating JUNV from neurological tissues, which may explain the persistence of neurological symptoms in some animals. This is well known for IgG treatment in animal models and human patients where systemic disease is well controlled, but neurological complications occur often in survivors (33–35). This is one of the hurdles for the therapy against South American hemorrhagic fevers since they cause neurological symptoms which are rarely observed in LF (36, 37). To improve the treatment efficacy of compound #261, especially for therapy against South American hemorrhagic fevers, it would be necessary to modify the compound or the route of administration for better pharmacokinetics in neurological tissues, or to combine it with a treatment for neurological symptoms.

Compound #261 is not effective for MACV and GTOV. This would be due to the amino acid differences around the active site in L proteins (18, 38). Therefore, through expanded screening efforts, we additionally identified CENi candidates with antiviral activity against MACV and GTOV. These findings further support the potential of CENi compounds as promising antiviral drug candidates for pan-arenavirus therapy, although further screening or modification of the compounds or a mixture of multiple compounds is needed to cover all pathogenic arenaviruses. Importantly, CENi compounds act through a mechanism distinct from other antiviral candidates for arenavirus infections, such as the nucleotide analog Favipiravir (28, 39), or entry inhibitors (40, 41), indicating the potential of combinational therapies against hemorrhagic fevers caused by arenavirus infections.

## MATERIALS AND METHODS

### Cell and virus

Vero and Vero E6 cells purchased from the American Type Culture Collection were maintained in Dulbecco’s modified Eagle’s medium (DMEM) supplemented with 10% heat-inactivated fetal bovine serum (FBS), 100 U/mL penicillin-streptomycin at 37°C in 5% CO_2_. LASVs (Pinneo, Sauerwald, and Ojoko), JUNV (Romero), MACV (Chicava), GTOV (S-26764 and VINH-95551), SABV (SPH114202), and CHAPV (200401071) were provided by Tomas G. Ksiazek at the University of Texas Medical Branch (UTMB). LASV (LF2384), which belongs to lineage IV, was isolated from a non-fatal human LF case during a 2012 outbreak in Sierra Leone (20, 42). The virus was propagated in Vero or Vero E6 cells, and virus-containing cell culture supernatant was stored in a −80°C freezer until use. All work with infectious arenaviruses was performed in the biosafety level 4 (BSL-4) facilities in the Galveston National Laboratory (GNL) at UTMB in accordance with institutional guidelines.

### Virus titration

Virus titers were determined by plaque assay as described elsewhere (32, 43). Briefly, confluent monolayers of Vero or Vero E6 cells in 12-well plates were inoculated with 100 µL of 10-fold diluted virus and incubated for 45 min at 37°C in CO₂ incubator. After washing out the inoculum, each well was overlaid with minimum essential medium containing 2 or 5% FBS, 100 U/mL penicillin-streptomycin, and 0.6% tragacanth or 1% Avicel RC-591 NF and further incubated for 5–10 days. The cells were then fixed with 10% Formalin and stained with crystal violet. Viral titers were represented as PFU. The condition of plaque assay for each arenavirus was summarized in Table 1.

**TABLE 1:** Plaque assay condition for each arenavirus

Virus	Strain	Cell	Overlay	Incubation period
Lassa virus	Pinneo	Vero	2% FBS and tragacanth	5–6 days
	Sauerwald	Vero	2% FBS and tragacanth	5–6 days
	Ojoko	Vero	2% FBS and tragacanth	5–6 days
	LF2384	Vero	2% FBS and tragacanth	5–6 days
Junin virus	Romero	Vero	2% FBS and tragacanth	6–7 days
Machupo virus	Chicava	Vero	2% FBS and tragacanth	6–7 days
Guanarito virus	S-26764	Vero E6	5% FBS and Avicel	10 days
	VINH-95551	Vero	2% FBS and tragacanth	6–7 days
Sabia virus	SPH114202	Vero	2% FBS and tragacanth	7–8 days
Chapare virus	200401071	Vero E6	5% FBS and Avicel	10 days

### Compounds

**Fig 7:**
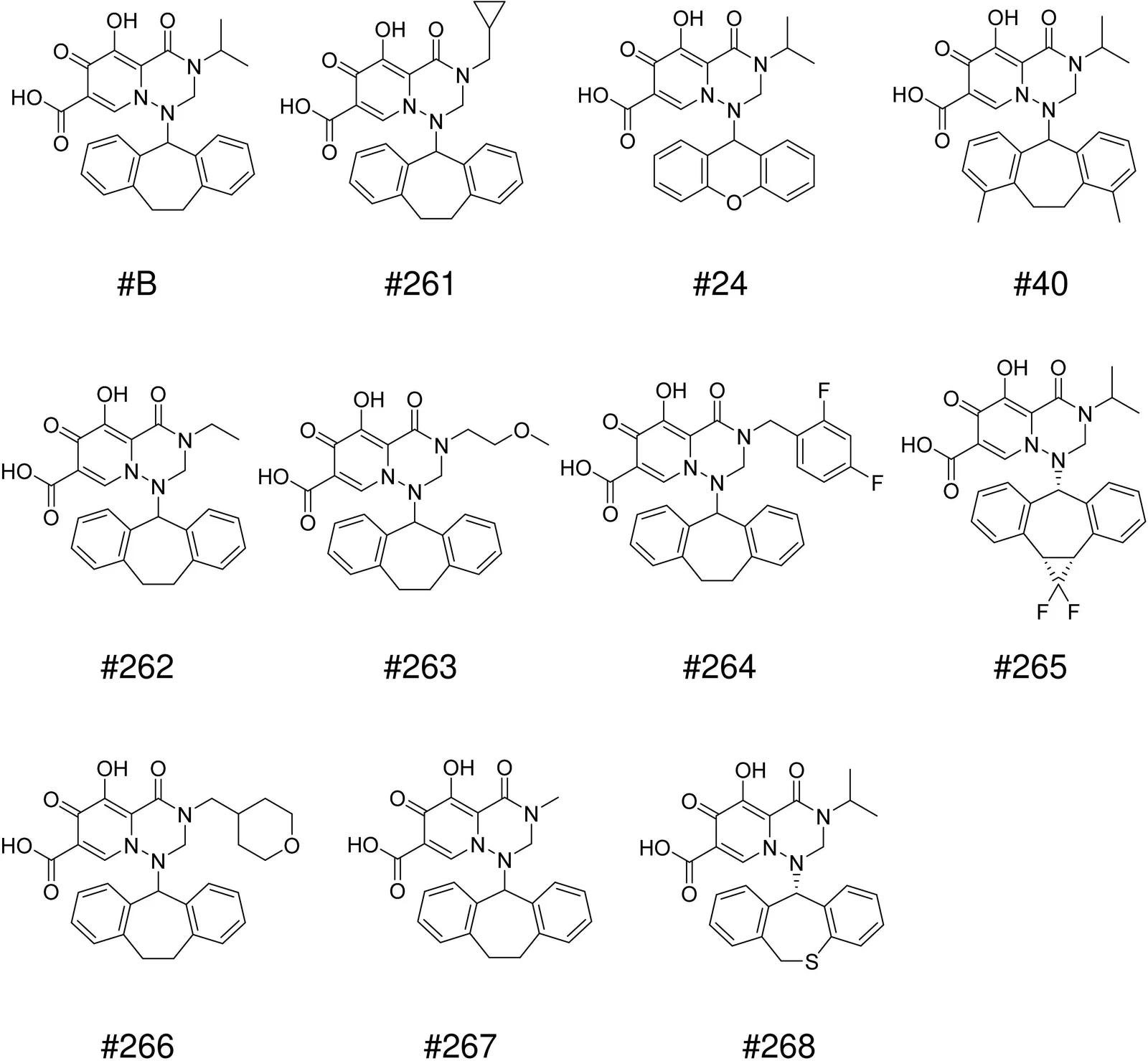
Chemical structures of CENi compounds in this study.

All CENi compounds, compound B (18), #261–268 (38), and #1–52 were supplied by Shionogi & Co., Ltd. Ribavirin was purchased from Wako. LHF-535 was purchased from Shanghai Chemhere Co., Ltd. For *in vitro* experiments, all compounds were dissolved in dimethyl sulfoxide (DMSO) and diluted in 2% FBS-MEM to yield 1% DMSO as a final concentration for antiviral assays. For *in vivo* experiments, compound #261 was dissolved in polyethylene glycol 400 (PEG-400), and ribavirin was dissolved in phosphate-buffered saline (PBS) with 5% DMSO. Chemical structures of compounds #B, #261, #24, and #40 are shown in Fig. 7.

### Virus replication inhibition assay *in vitro*

Vero or Vero E6 cells cultured in 12-well plates were infected with each virus at a multiplicity of infection (MOI) = 0.01 (LASV, LUJV, JUNV, MACV, GTOV, SABV, and CHAPV) or MOI = 0.1 (GTOV) and incubated for 1 h at 37°C. Followed by washing with DMEM containing 2% FBS three times, infected cells were cultured in DMEM, 2% FBS with various doses of the compound at 37°C. Cell culture supernatant samples were collected at 72 h post-infection and titrated by plaque assay. Each compound was dissolved and serially diluted in DMSO and then diluted in DMEM containing 2% FBS to a final concentration of 1% DMSO. The half-maximal inhibitory concentration (IC_50_) was determined by four-parameter logistic curve fitting.

### Cell viability assay

Vero cells seeded in 96-well plates were treated with 4-fold serial dilutions of compounds starting at 250 µM. Each compound was dissolved and serially diluted in DMSO and then diluted in DMEM containing 2% FBS to a final concentration of 1% DMSO. After incubation for 72 h at 37°C, cell viability was evaluated by CellTiter-Glo (Promega) according to the manufacturer’s instructions. Relative luminescence units (RLUs) were measured using a GloMax 96 luminometer (Promega), and cell viability (%) was calculated by normalizing the RLUs of each sample to those of untreated control cells, which were defined as 100% viability. If cell viability did not fall below 50% at the highest concentration tested, the CC₅₀ value was defined as >250 µM.

### Treatment study in guinea pigs infected with LASV or JUNV

Six- to eight-week-old Hartley guinea pigs were purchased from Charles River Laboratories. All animals were housed in animal biosafety level 2 (ABSL-2) and ABSL-4 facilities in the GNL at UTMB. Before being transferred to ABSL-4, the animals were maintained in ABSL-2 facility for at least 7 days following their delivery. Prior to virus challenge, the animals were maintained in ABSL-4 for at least 3 days to facilitate acclimation. Animal identification and measuring of body temperature were performed with subcutaneously implanted BMDS IPTT-300 transponders and a DAS-8027 transponder reader (Bio Medic Data Systems). Guinea pigs (*n* = 5) were intraperitoneally inoculated with 10^3^ PFU of LASV (LF2384) or JUNV (Romero) in 100 μL of PBS. Subsequently, the guinea pigs received treatment with compound #261, ribavirin, or respective vehicle controls, starting at 2 days post-inoculation (d.p.i.) and continuing for 14 days via subcutaneous injection. Animals were humanely euthanized once they showed neurological symptoms, could not access their food or water, exhibited a body temperature below 32°C (hypothermia), or lost more than 20% of their body weight. The clinical index was calculated as the mean of the animals (normal = 0; one visible symptom = 1; more than two visible symptoms = 2; and dead/euthanized = 3). In addition, guinea pigs were intraperitoneally inoculated with 10^2^ PFU of LASV (LF2384) and then treated with compound #261 (*n* = 5) or the vehicle control (*n* = 4) daily via subcutaneous injection, starting at 2 d.p.i. The organ (brain, lung, liver, spleen, and kidney) and serum samples were collected at 11 d.p.i. to assess the virus dissemination and complete blood count.

### Complete blood count

Whole-blood samples were collected in EDTA tubes from LASV-infected guinea pigs at 11 d.p.i. or the end of the study (28 d.p.i.). Complete blood counts were measured using a VETSCAN HM5 (ABAXIS) according to the manufacturer’s instructions.

### Plaque reduction neutralizing test

Neutralizing antibody was detected by plaque reduction neutralizing test with collected serum samples from survivors infected with LASV at the end of the study. LASV (LF2384) was diluted with 2% FBS DMEM to yield 80 PFU/0.1 mL and mixed with the same volume of serially diluted heat-inactivated serum. After incubation for 30 min at 37°C, the mixture was inoculated into a monolayer of Vero cells in 12-well plates and incubated in CO_2_ for 30 min at 37°C. After washing inoculum out, MEM with 0.6% Tragacanth (Sigma) and 2% FBS was added as an overlay. After incubation for 5–6 days, cells were fixed with 10% formalin, and plaques were visualized by crystal violet staining. Antibody titers were expressed as 50% plaque reduction neutralizing titers (PRNT_50_), which is the lowest dilution that demonstrates greater than 50% neutralization relative to the control.

### Statistical analysis

Statistical analyses were performed with GraphPad Prism Software (ver. 10.2.2). Log-rank test followed by Bonferroni correction was used for analysis of differences in survival compared to the vehicle control group. Statistical differences for body weight/temperature change were calculated by two-way analysis of variance (ANOVA), followed by Dunnett’s multiple comparison test. Statistical differences for platelet/white blood cell counts and PRNT_50_ were calculated by one-way ANOVA, following Dunnett’s multiple comparison test. Statistical differences for virus titers were calculated by Welch’s *t*-test.

## Data Availability

All data are available in the main text or the supplemental materials.

## SUPPLEMENTAL MATERIAL

Supplemental tables mbio.00880-26-s0001.xlsx Tables S1 to S6.

ASM does not own the copyrights to Supplemental Material that may be linked to, or accessed through, an article. The authors have granted ASM a non-exclusive, world-wide license to publish the Supplemental Material files. Please contact the corresponding author directly for reuse.
